# Severe Clinical Manifestation of a Salt Wasting Form of Congenital Adrenal Hyperplasia Harboring a Complex Genotype

**DOI:** 10.1155/crie/1599576

**Published:** 2025-12-08

**Authors:** Irene Fylaktou, Vasiliki Rengina Tsinopoulou, Amalia Sertedaki, Christina Kanaka-Gantenbein, Assimina Galli-Tsinopoulou

**Affiliations:** ^1^ Division of Endocrinology, Diabetes and Metabolism ‘Aghia Sophia’ Children’s Hospital ENDO-ERN Center for Rare Paediatric Endocrine Diseases, First Department of Pediatrics, Medical School, National and Kapodistrian University of Athens, ‘Aghia Sophia’ Children’s Hospital, Athens, Greece, uoa.gr; ^2^ 2nd Department of Paediatrics, School of Medicine, Faculty of Health Sciences, Aristotle University of Thessaloniki, AHEPA University General Hospital, Thessaloniki, Greece, auth.gr

## Abstract

**Background:**

21‐hydroxylase deficiency (21‐OHD) represents the most common form of congenital adrenal hyperplasia (CAH) caused by pathogenic variants in the *CYP21A2* gene. To date the molecular analysis of the *CYP21A2* gene is based on the selective *CYP21A2* gene amplification followed by Sanger sequencing. Herein we present the clinical manifestations, hormonal profile, and application of different molecular strategies to accurately investigate the *CYP21A2* gene in a newborn presenting with the salt wasting (SW) form of CAH.

**Methods:**

The patient, his parents, and the paternal grandparents underwent *CYP21A2* genotyping employing Sanger sequencing and multiplex ligation‐dependent probe amplification (MLPA). Furthermore, the patient and his parents underwent a more extended protocol to determine the location of the pathogenic variants identified.

**Results:**

The newborn carried three *CYP21A2* gene copies (two paternal and one maternal), each of them harboring a pathogenic variant, which is in concordance with the clinical manifestations of the SW form of CAH.

**Conclusion:**

The molecular investigation of the proband presenting with the SW form of CAH revealed a complex genotype that could only be determined by employing different molecular strategies. Laboratories should be aware of the possibility of complex genotypes in the *CYP21A2* gene and employ different protocols to avoid the possibility of a genetic misdiagnosis.

## 1. Introduction

Congenital adrenal hyperplasia (CAH) is an autosomal recessive disorder with the majority of cases (90%–95%) to be caused by defects in the 21‐hydroxylase enzyme leading to CAH due to 21‐hydroxylase deficiency (21‐OHD) [1]. There are two forms of 21‐OHD the severe classic (C) and the milder non classic form (NC). The classic form presents at birth with severe clinical manifestations (atypical genitalia in females, enlarged penis in males) and is further divided in the classic salt wasting (SW) form that manifests with severe salt loss, failure to thrive, potentially fatal hypovolemia, shock (due to both cortisol and aldosterone insufficiency) and the classic simple virilizing form (SV) that manifests with signs of excess androgen production without salt loss due to sufficient aldosterone production. The NC form presents a variety of milder clinical manifestations (ranging from asymptomatic cases to premature adrenarche, hirsutism, acne, advanced bone age, and menstrual irregularities). The genetic cause for 21‐OHD is attributed to pathogenic variants in the *CYP21A2* gene. Gene conversion events between the pseudogene and the *CYP21A2* gene account for 75% of pathogenic variants identified [2]. Gene deletions and CYP21A1P‐CYP21A2 chimeras, resulting from misalignment of the sister chromatids and unequal crossovers, correspond to the 20%–25% of 21‐OHD alleles [1]. The remaining 5%–10% of the 21‐OHD pathogenic variants represent novel or rare variants while de novo aberrations have also been described with an incidence of 1%–2% [1].

The *CYP21A2* gene is located on the short arm of chromosome 6 (6p21.31), within the major histocompatibility complex (MCH) class III region, in a genetic unit known as the RCCX fragment [3, 4]. The RCCX fragment comprises the serine/threonine kinase (RP), the complement C4, the *CYP21A2* gene and the tenascin gene (TNX) [4]. This represents the monomodular haplotype. To date, different haplotypes have been described originating from the different copy number of the RCCX fragment [5, 6]. Different copies of the RCCX fragment result in different copies in the *CYP21A2* gene thus rendering the molecular diagnosis of the *CYP21A2* challenging. It has been frequently observed that in the presence of two copies of the *CYP21A2* gene on the same allele, the *CYP21A2* gene located downstream of the *TNXA* gene preserves the wild type sequence or carries one or more deleterious variants while the *CYP21A2* gene downstream of the *TNXB* gene can also preserve the wild type sequence or may harbor the p.Gln319Ter pathogenic variant [7].

Herein, we report the clinical manifestation, the hormonal profile and the molecular investigation of the *CYP21A2* gene in a newborn with the SW form of CAH harboring a complex genotype.

## 2. Case Presentation

### 2.1. Clinical Presentation

An 11‐day‐old newborn male was brought by his parents to the Emergency Department due to weakness, lethargy, and refusal to eat. The parents reported two episodes of vomiting; 48 and 24 h before hospital admission.

The neonate, the first child of a non‐consanguineous marriage, was conceived by in vitro fertilization (IVF), and was born after an uneventful full‐term pregnancy (39 weeks) with BW: 3.350 g (50th–75th percentile), BL: 51 cm (50th–75th percentile) and head circumference 35.5 (95th percentile). No adverse events were reported after birth. The family history was unremarkable. During neonatal screening for metabolic diseases on the 3rd day of life, increased levels of 17‐hydroxyprogesterone (17‐OHP:17.2 ng/mL) were detected.

On admission the neonate presented with decreased skin turgor, severe dehydration, cyanosis, poor peripheral circulation, hypothermia, hypotonia, bradycardia, arrhythmia, and severe hypotension. Weight loss was observed (weight: 3.140 g) as well as jaundice was noted with an icteric skin coloration and conjunctiva of the eyes. Further clinical examination revealed male genitalia, with testes located in a mildly hyperpigmented scrotum and 5 cm penile length (reference range to age: 3.5 ± 0.4).

Initial laboratory evaluation revealed life‐threatening hyperkalemia, hyponatremia, hypoglycemia, and metabolic acidosis (Table 1). The neonate presented an apneic episode and demonstrated severe electrocardiographic (EKG) abnormalities such as peaked T waves, flattering P wave and wide QRS complex, due to severe electrolyte disturbances, especially hyperkalemia.

**Table 1:** Laboratory findings of the patient on admission.

Laboratory tests	Patient’s value	Normal range
17‐OHP (ng/mL)	**17.2**	<6.3
Aldosterone (pg/mL)	**13.7**	50–1750
Renin (pg/mL)	**572.64**	78.8 ± 9.6
DHEA‐S (μg/dL)	**19.1**	500–11,100
Testosterone (ng/dL)	130.2	1–177
Δ4‐Androstenedione (ng/mL)	**635**	18–80
ACTH (pg/mL)	**1174**	7.2–63.6
Glucose (mg/dL)	**56**	70–110
Potassium (mEq/L)	**10**	3.2–5.8
Sodium (mEq/L)	**120**	137–150
Values at metabolic acidosis		
pH	7.31	—
cHCO_3_ (mmol/L)	13.6	—
Base excess (mmol/L)	−15.1	—

*Note:* Values outside the normal range are depicted in bold.

Initial management of dehydration included intravenous fluids and correction of life‐threatening hyperkalemia with polystyrene sulfonate enema (1 g/kg) during the first 24 h of admission. The increased 17‐OHP in combination with the severe clinical manifestations and relevant laboratory findings (Table 1) evoked the diagnosis of the SW form of classic CAH, especially combined with the correction of dehydration and hyperkalemia, when stress dose intravenous hydrocortisone was administered. Poor peripheral circulation and the inability to venipuncture did not allowed for the intravenous administration of calcium or insulin for the treatment of hyperkalemia. After 48 h, electrolytes and glucose values improved and normalization occurred after 72 h; the patient was then placed on oral substitution therapy with hydrocortisone and flurohydrocortisone. In addition, NaCl (1 g/day) was added, divided in six doses just before meals.

The newborn was discharged after 1 week in excellent general condition with completely normal values of electrolytes and glucose and normal ECG findings.

The infant, now 12 months old, has normal physical and psychomotor development (both weight and height are above the 95th percentile on growth charts) and normal dentition, while there were no reports of infections or hospitalizations. He has undergone the age‐appropriate vaccinations without any side‐effects and the substitutive dose of hydrocortisone and flurohydrocortisone has been properly adjusted according to his body surface area. Supplementary oral NaCl administration has been discontinued at the age of 12 months since the infant is on complete enteral feeding with solid foods. His routine laboratory examinations, including 17‐OHP, ACTH, and Δ4‐Androstenedione values, were normal during the whole follow‐up period.

### 2.2. Molecular Analysis

#### 2.2.1. Subjects

Blood samples of the index patient, his parents, and the paternal grandparents were sent to the Laboratory of Molecular Endocrinology, Division of Endocrinology, Diabetes, and Metabolism, Aghia Sophia ENDO‐ERN Center for Rare Paediatric Endocrine Diseases for molecular analysis of the *CYP21A2* gene. Written informed consent was obtained from the parents for publication of this case report.

#### 2.2.2. DNA Extraction

Genomic DNA was isolated from peripheral blood samples employing the QIAamp DNA Blood Mini Kit (Qiagen, Hilden, Germany) according to the manufacturer’s instructions.

#### 2.2.3. *CYP21A2* Gene Sequencing and Multiplex Ligation‐Dependent Probe Amplification (MLPA) Analysis

Bidirectional Sanger sequencing of the exons and introns of *CYP21A2* gene was carried out after selective amplification against its pseudogene *CYP21A1P*, as previously described [8]. Analysis of the *CYP21A2* gene was based on the reference gene NG_007941.2 and transcript NM_000500.9.

MLPA employing the P050‐D1 CAH kit (MRC Holland, Amsterdam, The Netherlands) was carried out in all subjects for the identification of duplication/deletion of the *CYP21A2* gene.

#### 2.2.4. Detection of the *CYP21A2* Gene Downstream of the *TNXB* Gene

The complete *CYP21A2* gene downstream of the *TNXB* gene was amplified employing the CYP779‐F and TENA‐32F generating a 8.5 Kb fragment as previously described [9, 10]. This fragment was reamplified (nested PCR) with specific primers in order to investigate the distribution of the pathogenic variants identified in the proband and his parents. Bidirectional sequencing was performed in the amplified fragments with the use of internal primers employing the Big Dye Terminator Cycle sequencing kit and ABI 3500 Genetic Analyzer (PE Applied Biosystems, Foster City, CA).

#### 2.2.5. Detection of the *CYP21A2* Gene Downstream of the *TNXA* Gene

The complete *CYP21A2* gene downstream of the *TNXA* gene was amplified with the primers CYP779‐F and XA‐36F generating a 6.1 Kb product [11]. The PCR mixture contained 2 μM of dNTPs mix, 5 μL of 5xQ5 reaction buffer, 2 U/μL of Q5 HiFi Taq (New England BioLabs), 5xQ5 High GC Enhancer, 7.5 μM of each primer, and 150 ng of genomic DNA at a final volume of 25 μL. Amplification was performed after initial denaturation at 98°C of 3 min followed by 35 cycles of denaturation (98°C, 10 s), annealing (68°C, 30 s), extension (72°C, 3 min), with a final extension at 72°C for 1 min. In order to differentiate the *CYP21A2* gene from its pseudogene specific internal primers (nested PCR) were employed, and a negative control containing 2 copies of the *CYP21A2* gene was used in order to ensure the amplification of the *CYP21A2* gene. Bidirectional sequencing was performed only for the verification of pathogenic variants identified in the amplified fragments employing the Big Dye Terminator Cycle sequencing kit and ABI 3500 Genetic Analyzer (PE Applied Biosystems, Foster City, CA).

### 2.3. Results

Sanger sequencing revealed that our patient harbored the pathogenic variants, c.293‐13C>G (I2splice) and p.Gln319Ter as well as the SNPs c.293‐79G>A (rs114414746) and c. ^∗^12C>T (rs150697472), all in heterozygosity. The presence of the two SNPs is indicative for the presence of two copies of the gene in one allele and that the *CYP21A2* gene downstream of the *TNXB* gene will harbor the pathogenic variant p.Gln319Ter. The MLPA analysis confirmed the presence of three copies of the *CYP21A2* gene in the patient (Figure 1).

Figure 1MLPA representative ratio charts of the patient and his parents. The patient has three copies of the *CYP21A2* gene and carries the A allele in position c.293‐13 in one copy of the *CYP21A2* gene indicating that the other two copies harbor the G allele in position c.293‐13. The mother has two copies of the *CYP21A2* gene and carries the A allele in position c.293‐13 in one copy of the *CYP21A2* gene indicating that the other copy of the *CYP21A2* harbors the G allele in position c.293‐13. The father harbors three copies of the *CYP21A2* gene and carries the A allele in position c.293‐13 in the two copies of the *CYP21A2* gene indicating that the third copy of the *CYP21A2* gene harbors the G allele in position c.293‐13.

**Figure:**
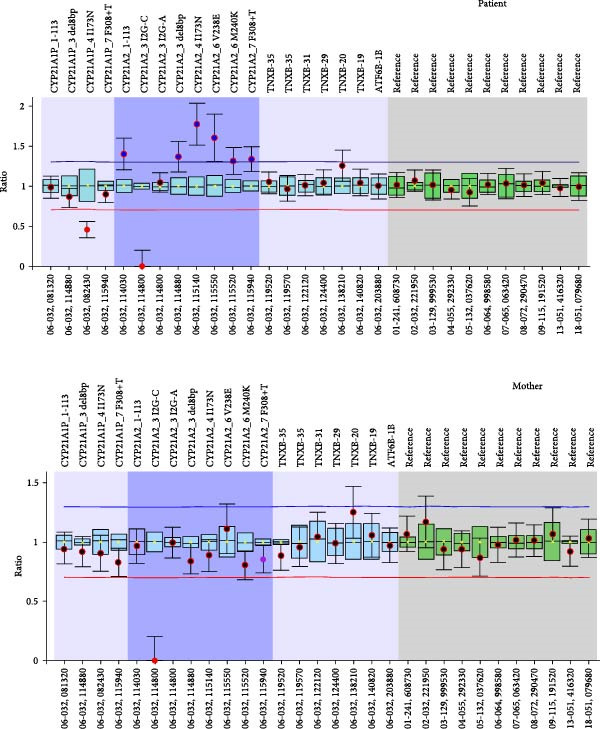

**Figure:**
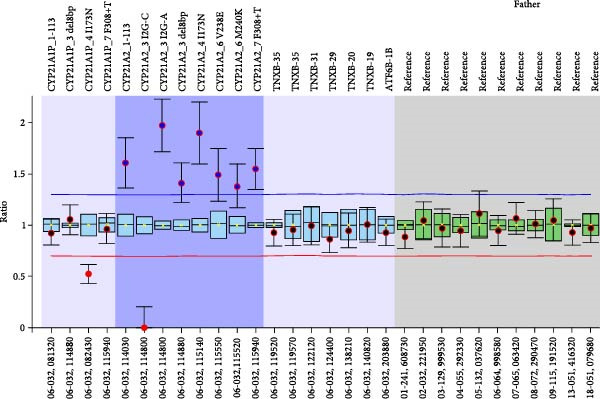

Sanger sequencing of the parents revealed that (a) the mother was heterozygous for the c.293‐13C>G and (b) the father was heterozygous for the pathogenic variants c.293‐13C>G and p.Gln319Ter and the SNPs c.293‐79G>A (rs114414746) and c. ^∗^12C>T (rs150697472). The MLPA analysis of the parents revealed that the mother carried two copies of the *CYP21A2* gene, while the father carried three copies of the *CYP21A2* gene (Figure 1).

Father’s parental samples were analyzed in order to delineate the distribution of the identified pathogenic variants in association with the three copies of the *CYP21A2* gene. Sanger sequencing and MLPA analysis revealed that the duplicated *CYP21A2* gene together with the pathogenic variant p.Gln319Ter were maternally inherited, whereas the pathogenic variant c.293‐13C>G was a de novo aberration.

To investigate the distribution of the 2 pathogenic variants in the proband, we employed the extended protocol described above, which amplified and sequenced the *CYP21A2* gene downstream of the *TNXB* in the proband and both parents as well as the *CYP21A2* gene downstream of the *TNXA* in the proband and his father.

The analysis revealed that the proband carried the maternal c.293‐13C>G and the paternal p.Gln319Ter variants along with the paternal SNP c.293‐79G>A (rs114414746) in heterozygosity in the *CYP21A2* gene downstream of the *TNXB* and the paternal c.293‐13C>G along with the SNP c. ^∗^12C>T (rs150697472) in the *CYP21A2* gene downstream of the *TNXA* (Figure 2A,B). The proband was therefore considered to be a compound heterozygote for the SW form of CAH, while the father is considered to be a heterozygote for the SW form of CAH.

Figure 2Schematic representation of the family’s genotypes. (A) Schematic representation of the mother’s and father’s genotype. (B) Schematic representation of patient’s genotype.

**Figure:**
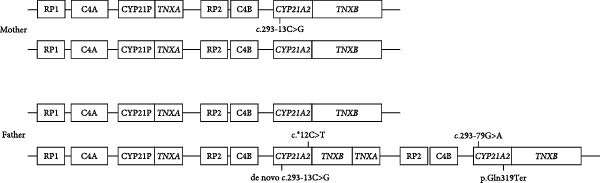
(A)

**Figure:**
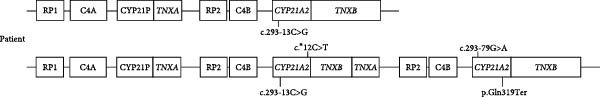
(B)

## 3. Discussion

We present a newborn with the SW form of CAH bearing a complex genotype consisting of three *CYP21A2* gene copies (two paternal and one maternal), each one of them harboring a pathogenic variant. Particularly the maternal allele and one of the two paternal *CYP21A2* gene copies harbor the c.293‐13C>G, while the second paternal *CYP21A2* gene harbors the p.Gln319Ter.

Our patient’s genotype is rare and could be easily misdiagnosed due to the presence of the same pathogenic variant (c.293‐13C>G) carried by both parents and the existence of three copies of the *CYP21A2* gene. To date, the comprehensive genotyping of the *CYP21A2* gene, comprising Sanger sequencing followed by MLPA, remains the gold standard for 21‐OHD genetic diagnosis [12]; however, it cannot always elucidate rare and complex genotypes. In this case, the comprehensive *CYP21A2* genotyping revealed the presence of the c.293‐13C>G and p.Gln319Ter in heterozygosity but their allocation in the three gene copies present was not easily identifiable. To determine precisely the genotype of our patient, it was crucial to employ the extended protocol described [5, 6, 13, 14].

To date, different haplotypes have been reported involving the RCCX fragment [6, 14–17]. More precisely, in the European population three different haplotypes have been described; the monomodular (one copy of the RCCX fragment), the bimodular (two copies of the RCCX fragment) and the trimodular (three copies of the RCCX fragment) with a prevalence of 15%, 75% and 10%, respectively [6, 7, 18]. The trimodular haplotype, involving two copies of the *CYP21A2* gene along with the p.Gln319Ter, usually in linkage disequilibrium with two specific SNPs (rs150697472 and rs114414746) [10, 17, 19], is known to be present in less than 2% in the general population [17]. In our population, the trimodular haplotype is found in a slightly higher frequency emphasizing the importance of identifying the correct copy number in the *CYP21A2* gene and particularly in cases, such as the one presented herein, where the p.Gln319Ter is present, as it may alter genetic diagnosis [8, 20]. The presence of the p.Gln319Ter in heterozygosity and the existence of the *CYP21A2* gene in each allele leads to the genetic diagnosis of a heterozygote for the SW form of CAH. The presence of three copies of the *CYP21A2* gene (trimodular haplotype) along with the p.Gln319Ter complicates genetic diagnosis, since the exact position of p.Gln319Ter with respect to the copies of the *CYP21A2* gene is not defined. If the p.Gln319Ter is present on the allele bearing the two copies of the *CYP21A2* gene, then the individual is not considered as heterozygote since both alleles have one functional *CYP21A2* gene. In the case where the p.Gln319Ter is present on the allele bearing one copy of the gene, then the individual is considered to be heterozygote for the SW form of CAH, since there is only one allele with functional *CYP21A2* gene.

Furthermore, de novo aberrations are known to occur due to unequal crossing over between chromosomes at the RCCX fragment [1, 2, 21, 22]. It has been shown that gene duplications particularly of the maternal allele predisposes to de novo aberrations in the offspring [23]. This is supported by our case since one of the two fathers’ maternally inherited alleles was harboring the de novo pathogenic variant c.293‐13C>G.

The clarification of complex genotypes is important for the patient and his family, as well as for correct genetic counseling. In our case, the duplication of the *CYP21A2* gene and the presence of the same pathogenic variant in both parents resulted in a complex genotype, that could not have been defined by employing routine diagnostic methods.

Although accurate genotype definition confirms clinical diagnosis and has no effect on treatment, care, and prognosis, nevertheless, it is important for family planning and genetic counseling. More precisely, genetic diagnosis provides the ability of prenatal testing allowing for detailed early diagnosis and prevention of the complications of SW form of CAH, in cases where the fetus is affected.

The identification of our patient’s genotype confirmed the clinical diagnosis sparing the patient and his family of a genetic odyssey and uncertainty in their family planning although not impacting on his treatment and care. Nevertheless, genetic diagnosis provided the ability for proper genetic counseling and planning to the patient’s family.

In conclusion, we present a neonate with clinical manifestations of SW form of CAH and a rare and complex genotype of *CYP21A2* gene, which could only be determined after the employment of an extended diagnostic protocol. Although, such cases are rare, laboratories should be aware that complex genotypes, might occur, and the appropriate protocols should be employed, to avoid genetic misdiagnosis and allow appropriate genetic counseling of the affected family.

## Consent

Written informed consent was obtained from the parents for publication of this case report.

## Conflicts of Interest

The authors declare no conflicts of interest.

## Author Contributions

Concept and design: Irene Fylaktou, Vasiliki Rengina Tsinopoulou, Amalia Sertedaki, Christina Kanaka‐Gantenbein, and Assimina Galli‐Tsinopoulou. Drafting of the manuscript: Irene Fylaktou and Vasiliki Rengina Tsinopoulou. Critical review of the manuscript for important intellectual content: Irene Fylaktou, Vasiliki Rengina Tsinopoulou, Amalia Sertedaki, Christina Kanaka‐Gantenbein, and Assimina Galli‐Tsinopoulou. Acquisition, analysis, or interpretation of data: Irene Fylaktou, Vasiliki Rengina Tsinopoulou, Amalia Sertedaki, Christina Kanaka‐Gantenbein, and Assimina Galli‐Tsinopoulou. Supervision: Amalia Sertedaki, Assimina Galli‐Tsinopoulou, and Christina Kanaka‐Gantenbein. Irene Fylaktou and Vasiliki Rengina Tsinopoulou contributed equally in this work.

## Funding

This research received no external funding.

## Data Availability

Data available upon request due to privacy/ethical restrictions.
